# Usage of Probabilistic and General Regression Neural Network for Early Detection and Prevention of Oral Cancer

**DOI:** 10.1155/2015/234191

**Published:** 2015-06-15

**Authors:** Neha Sharma, Hari Om

**Affiliations:** ^1^Dr. D.Y. Patil Institute of Master of Computer Applications, Akurdi, Savitribai Phule Pune University, Maharashtra 411007, India; ^2^Computer Science and Engineering Department, Indian School of Mines, Dhanbad, Jharkhand 826004, India

## Abstract

In India, the oral cancers are usually presented in advanced stage of malignancy. It is critical to ascertain the diagnosis in order to initiate most advantageous treatment of the suspicious lesions. The main hurdle in appropriate treatment and control of oral cancer is identification and risk assessment of early disease in the community in a cost-effective fashion. The objective of this research is to design a data mining model using probabilistic neural network and general regression neural network (PNN/GRNN) for early detection and prevention of oral malignancy. The model is built using the oral cancer database which has 35 attributes and 1025 records. All the attributes pertaining to clinical symptoms and history are considered to classify malignant and non-malignant cases. Subsequently, the model attempts to predict particular type of cancer, its stage and extent with the help of attributes pertaining to symptoms, gross examination and investigations. Also, the model envisages anticipating the survivability of a patient on the basis of treatment and follow-up details. Finally, the performance of the PNN/GRNN model is compared with that of other classification models. The classification accuracy of PNN/GRNN model is 80% and hence is better for early detection and prevention of the oral cancer.

## 1. Introduction

The oral tumor is one of the ten most incessant diseases worldwide and its rate of occurrence is increasing in every decade. Two decades back, the yearly occurrence of the oral cancer was over 3,00,000 cases [1], which went up with occurrence of 5,75,000 new cases in last decade at global level [2]. More latest study indicates that the oral cancer related mortality has declined worldwide from 3,20,000 deaths to approximately 2,00,000 deaths in less than half decade due to improved infrastructure of the health system [3, 4]. The study shows that the developing countries have the highest rate of oral cavity cancer, whereas the developed countries have the lowest rate of oral cavity cancer, for both males and females [5]. The age-adjusted rates of oral tumor differ from over 20 for every 1,00,000 population in India to 10 for every 1,00,000 in the US and less than 2 for every 1,00,000 in the Middle East [4, 6]. It is clearly evident that there is a huge contrast in the rate of oral tumor in different regions in the world. In the US, oral cavity malignancy is only about 3% of malignancies, whereas, in India, it accounts for over 30% of all growths [6]. The head and neck cancers are the sixth common malignancy that is the major cause of cancer morbidity and mortality worldwide. In India, the cancers of head and neck comprise approximately 24.1% of total cancers reported at Tata Memorial Centre, Mumbai, and out of them about 3.2% are from the oral cavity [7] that ranks among the top three types of cancer in the country [8]. It is of tremendous public health importance in India as it has been estimated that 83,000 new oral cancer cases [9, 10] and 46,000 deaths [4] occur here each year. The difficulty level is high because it is usually diagnosed at later stage, which results in low treatment outcomes and considerable high cost of treatment to the patients who cannot afford this type of treatment [11]. Hence, early diagnosis and treatment is one of the most important means to improve the patient's survival. Therefore, the objective of this paper is to introduce the assistance of data mining to medical fraternity, with a special focus on early detection and prevention of oral cancer in patients.

According to Fayyad et al. [12, 13], data mining can formally be defined as a process of extracting nontrivial and potentially useful information from the enormous datasets, providing explicit knowledge that has a readable form and can be used to diagnose, classify, or forecast problems [12–17]. In this paper, we intend to use classification technique of data mining approach. The classification model is built by using the probabilistic neural network and general regression neural network (PNN/GRNN). Though it is a very powerful model, yet it has not been used much in the past. Our endeavour is to build a probabilistic neural network and general regression neural network (PNN/GRNN) model for early detection and prevention of oral cancer. These models can be helpful to practitioners for the following decisions:

- To diagnose the malignant patients and the type of malignancy on the basis of demographic information, clinical symptoms, medical and personal history, and gross examination.
- To predict the stage and extent of oral cancer on the basis of symptoms which are confirmed with the help of relevant tests and investigations.
- To predict the survivability of patients after appropriate treatments and follow-ups.

**Figure 1:**
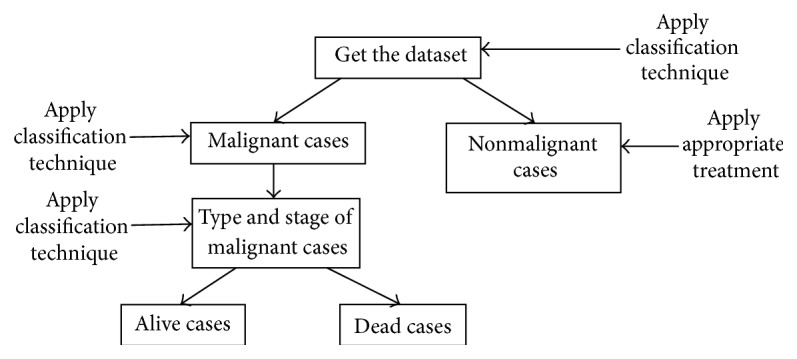
Use of PNN/GRNN classification for early detection and prevention of oral cancer.

The framework presented in Figure 1 is used to build PNN/GRNN model to classify malignant and nonmalignant cases, type of malignancy, and stage of malignancy and then all malignant cases are further classified to predict the survivability of the patients.

The rest of the paper is organized as follows: Section 2 discusses material and method adopted for the research and Section 3 covers brief discussion about the probabilistic neural network and general regression neural network. Sections 4 and 5 present the experimental results and discussions, respectively, to compare the performance of the PNN/GRNN model with that of the classification model developed previously.  Section 6 concludes the paper.

## 2. Material and Method

Identifying right source and selecting the relevant data are very important because the data mining learns and discovers the hidden patterns from the available data. Correctness and accuracy of data have a great impact on data mining analysis. Hence, a retrospective chart review of data from ENT (Ear-Nose-Throat) and Head-Neck Department of three Tertiary Care Hospitals of Pune, Maharashtra, India, has been carried out for data collection related to oral cancer. The records are fetched from the Cancer Registries of the Tertiary Care Centers, OPD (Out-Patient Department) datasheet which records the information regarding clinical details, personal history, habits, and so forth of the patients and from the archives of Departments of Histopathology, Surgery, and Radiology. The information was manually collected to complete the datasheet of the patients. The data of 1025 patients were collected in nonrandomized or nonprobabilistic method, as all the data in the registries for the period of five years have been considered. The dataset is based on the records of all the patients who have been reported with a lesion and treated at the centre from June 2004 to June 2009. The dataset thus collected has been transformed, cleaned, and integrated to make it ready for analysis and is presented in our previous paper [18].

Further, the dataset is reduced to perform classification at various levels using feature selection method which is one of the data reduction strategies. There are basically two categories of feature selection method: filter and wrapper [19]. The filter approach applies an independent test on data subset and has low computational cost and the wrapper approach applies a predetermined learning algorithm and requires great computational effort [20]. The wrapper is considered more reliable for data classification whereas the filter can be scaled up to high-dimensional datasets and it is computationally fast and independent of the learning algorithm [21, 22]. Our requirement is not only to select the subset of attributes, but also to know the ranking of the attributes so as to design a model for early detection and prevention of oral cancer. Therefore, we have applied filter method for attribute selection as it selects attributes using their characteristics.

WEKA3.7.9 has been used for feature selection. Attribute evaluation method is InfoGainAttributeEval which evaluates the worth of an attribute by measuring the information gain with respect to the class. Search method used is Ranker, which ranks the attributes by their individual evaluations. The information gain (IG) of an attribute *A* that belongs to dataset *D* is defined as follows:

(1) IGD,A=HD−∑v∈valuesAx∈D ∣ valuex,A=vD·Hx∈D ∣ valuex,A,

where *H*(*D*) is entropy, the expected information needed to classify a record in the dataset and it is defined as follows:

(2) $$\begin{array}{c} H\left(\;D\;\right)=-\overset{m}{\underset{i=1}{\sum }}p_{i}\log_{2}\left(\;p_{i}\;\right), \end{array}$$

where *p*_*i*_ is the probability that an arbitrary record in dataset *D* belongs to class *C*_*i*_ and is estimated by |*C*_*i*_, *D*|/|*D*|.

Feature selection approach is applied on the dataset of 1025 oral cancer patients, which initially had 35 attributes. Subsequently, subset of the attributes is chosen with the help of attribute reduction strategy. Then the PNN/GRNN model is built using the selected attributes and leave-one-out method is used for validation of the model. The validation method is a simple cross-validation that utilizes a single observation from the original sample as the validation data and the remaining observations as the training data. Further, we shall evaluate the performance of the model using estimation methods which are critical, as they provide awareness on the characteristics of design and help to refine the parameters in iterative manner of learning and for picking out one of the most suitable models so designed. The criteria used to estimate the model are sensitivity and specificity chart, which includes positive-negative ratio, accuracy, true positive, true negative, false positive, false negative, sensitivity, specificity, positive predictive value, negative predictive value, precision, recall, and area under ROC curve. The positive-negative ratio is the proportion of the positive category (malignant) and the negative category (benign) of the target variable. The accuracy of the model indicates the correctness of classification, which can be inferred from misclassification table. The patients who are predicted as malignant among the malignant patients are true positive (TP) cases. The patients who are predicted as nonmalignant among the nonmalignant patients are true negative (TN) cases. The patients who are predicted as nonmalignant among the malignant patients are false negative (FN) cases. The patients who are predicted as malignant among the nonmalignant patients are false positive (FP) cases. The sensitivity and specificity are calculated by using TP, TN, FP, and FN. The sensitivity means the probability that the algorithms can correctly predict malignancy and it is computed as Sensitivity = TP/(TP + FN). The specificity means the probability that the algorithms can correctly predict nonmalignancy and it is computed as Specificity = TN/(FP + TN) [23]. The positive predictive value (PPV) is the proportion of the patients with the disease, who are correctly predicted to have the disease. The PPV value for a perfect model would be 1.0. The negative predictive value (NPV) is the proportion of patients who do not have the disease and are correctly predicted as not having the disease. The NPV value for a perfect model would be 1.0. The precision is the proportion of cases selected by the model that have the true value; the precision is equal to PPV. The recall is the proportion of the true cases that are identified by the model; recall is equal to sensitivity. The *F*-measure is the harmonic mean of the precision and recall. It combines the precision and recall to give an overall measure of the quality of the prediction. The Receive Operating Characteristic (ROC) curve for a model is sensitivity in terms of one minus specificity. The ROC analysis is used for estimating the prediction ability of a model. The closer the value of the ROC is to 1.0, the better the model is. To build the classification model and analyze the data, a powerful statistical analysis program, DTREG tool, is used, which is a robust application that can easily be installed on any Windows system. DTREG reads comma separated value (CSV) data files that are easily created from almost any data [23].

## 3. Probabilistic Neural Network and General Regression Neural Network

**Figure 2:**
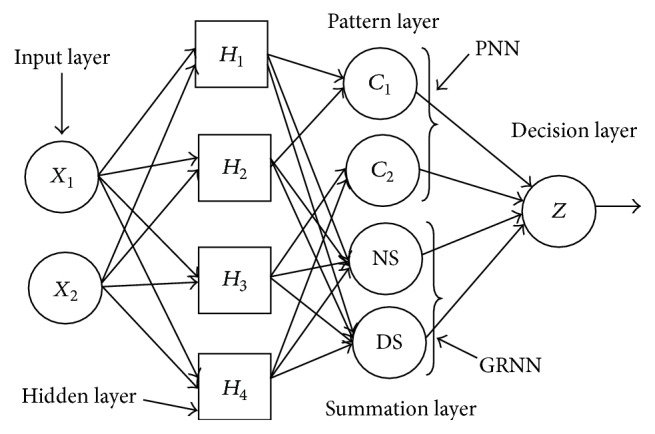
Architecture of probabilistic neural network and general regression neural network.

The probabilistic neural networks and general regression neural networks (PNN/GRNN) model consists of two networks that are integrated in a single architecture to handle different types of target variable. The probabilistic networks perform classification for categorical target variable and the general regression neural networks perform regression for continuous target variable. The PNN/GRNN model is usually much faster to train, more accurate, and relatively insensitive to outliers and generates accurate predicted target probability scores by approaching Bayes optimal classification. It is however slower in classifying new cases and requires more memory space to store the model [24–26]. The PNN/GRNN proposed by Specht [24] have four layers: input layer, hidden layer, pattern/summation layer, and decision layer, as shown in Figure 2. The input and hidden layers are same for the PNN and GRNN, but the pattern layer/summation layer and decision layer are different for the PNN and GRNN.

The input layer of the network has one neuron for each predictor variable (*X*_1_, *X*_2_,…), whose value is fed to each neuron in the hidden layer. Each neuron in the hidden layer stores the values of the predictor variables with its target value for each case in the training dataset (*H*_1_, *H*_2_,…). When the input values *X*_*i*_ are presented to the hidden layer, it computes the Euclidean distance from the neuron's central point for the test cases. This distance is then passed through the activation function, which is the RBF kernel function. The output of the hidden layer is fed to the next layer, which is different for PNN and GRNN. For PNN, this layer is known as pattern layer and there is one pattern neuron for each category of the target variable (*C*_1_, *C*_2_,…). The pattern neuron receives the weighted value of the training cases that belong to a particular target category as input from hidden layer. The pattern neurons add the values for the class they represent through the weighted vote for that category. For the GRNN, this layer is known as summation layer and there are only two neurons. One neuron is the denominator summation unit and the other is numerator summation unit (NS and DS). The denominator summation unit adds up the weight values coming from each of the hidden neurons. The numerator summation unit adds up the weight values multiplied by the actual target value for each hidden neuron [26]. The fourth layer of the network is the decision layer, which is again different for PNN and GRNN. For PNN, the decision layer compares the weighted votes for each target category accumulated in the pattern layer and uses the largest vote to predict the target category. For GRNN, the decision layer divides the value accumulated in the numerator summation unit by the value in the denominator summation unit and uses the result as the predicted target value [26].

## 4. Experimental Results

The file format of the database used to build the PNN/GRNN data mining model is comma separated values (.csv). There are total 1025 records that are described with the help of 35 attributes. The model is built with the help of DTREG tool.

### 4.1. Classification Model to Diagnose Malignancy and Benign Cases

12 predictor attributes have been selected by applying feature selection attribute for attribute reduction as explained in previous section and also by consulting the practitioners. The predictors are sex, socioeconomic status, clinical symptom, history of addiction, history of addiction1, comorbid condition, comorbid condition1, gross examination, site, predisposing factor, neck nodes, and tumor size. Attribute “Diagnosis” is selected as target variable, which may be malignant or benign. 75.5% of patients have been classified as malignant, whereas the 24.4% have been classified as benign. The malignant cases are treated as positive cases and the benign as negative. The classification accuracy of the model is 99.02% and sensitivity-specificity is also very high. The overall performance of the model for classification of malignant and benign cases on the basis of clinical symptoms is shown in Table 1.

### 4.2. Classification Model to Diagnose Type of Malignancy

The PNN/GRNN model has further been used to classify and diagnose the specific type of oral malignancy, using the same set of 12 attributes as predictor variables as given in previous section. The target variable is “Diagnosis,” which may be one of the various types of malignant tumor cases (acantholytic, adenocarcinoma, basaloid, lymphoepithelioma-like, plaque-like, sarcomatoid, squamous cell carcinoma, and verrucous) or benign cases. The performance of the model for classification of various types of malignant tumor cases on the basis of clinical symptoms is shown in Table 2. The overall accuracy of the model to predict the type of cancer for the training dataset is 93.85% and that for the validation dataset is 92.85%. The probability of various types of oral cancer that may occur is given in Table 3.

### 4.3. Classification Model to Diagnose the Stage of Malignancy

The next set of 6 attributes considered as predictors have been selected by applying feature selection attribute for attribute reduction as explained in previous section. These identified predictors are pertaining to the investigations (Diagnosis, LFT (Liver Function Test), FNAC of Neck Node, Diagnostic Biopsy, USG, and CT Scan/MRI) and the target variable is “Staging.” The PNN/GRNN model is used to predict the stage and the extent of the malignant cases. The performance of the model for prediction of stage of the oral cancer is presented in Table 4. The probability of stage I is 0.09%, stage II is 20.97%, stage IV is 43.02%, and stage N0 is 35.90%. The overall accuracy of the model for the training data and validation data is same, that is, 86.83%.

### 4.4. Classification Model to Diagnose Survivability of Oral Cancer Patients

Finally, on the basis of stage of cancer, the appropriate treatment and follow-ups are initiated and the survival rate of the patient is predicted by using the PNN/GRNN model as shown in Table 5. The set of 14 attributes considered as predictors are stage, surgery, radiotherapy, chemotherapy, 1st–5th follow-up symptoms, and 1st–5th follow-up examination, which have been selected by applying feature selection attribute for attribute reduction as explained in previous section, and the target variable is “Survival.”

The probability of dead cases is 40.2% and that of alive cases is 59.7%. The overall accuracy of the model to predict the survivability using 14 attributes is 69.95% for the training data as well as validation data. However, when 34 predictors were considered to predict the survivability, the classification accuracy was 80% for the training data and 73.76% for the validation data. Thus, the experimental results show that the PNN/GRNN data mining approach is appropriate for developing a model for early detection and prevention of oral cancer.

## 5. Discussions

Data mining has been used in healthcare for quite some time. However, its latest advanced techniques like neural networks have not been explored much for developing the decision making methods [27]. Bruins et al. [28] have used decision algorithm and decision tree to propose a model for developing and testing the evidence-based guidelines for pretherapy oral screening and dental management of patients with head and neck cancer. This model, tested by using a probabilistic sensitivity analysis with second-order Monte Carlo simulations (*n* = 10.000), reports that the decision tree and algorithm can be used for developing evidence based clinical guidelines. Rosmai et al. [29] study a fuzzy neural network model and fuzzy regression model to predict the likelihood of an individual to develop oral cancer based on the knowledge of their risk habits and demographic profiles at Oral Cancer Research and Coordinating Centre by using the sensitivity and specificity. The accuracy, sensitivity, and specificity for the fuzzy neural network model are reported as 59.9, 45.5, and 85.3; for clinicians prediction as 63.1, 54.2, and 78.6; and for fuzzy regression model as 67.5, 69.0, and 64.7, respectively. The model based results are better than that of the of oral cancer clinicians. Paper [30] discusses the fuzzy logic, fuzzy neural network, and fuzzy linear regression models to predict the oral cancer susceptibility. For 1-input and 2-input predictor sets, all three models have 64% prediction accuracy. For more number of inputs, for example, 3 inputs and 4 inputs, the prediction accuracies of both the fuzzy neural networks and fuzzy linear regression increase to 80%, while there is no change for fuzzy logic prediction. HariKumar et al. [31] analyse the classification accuracy of the TNM (tumour, lymph nodes, and metastasis) staging system along with that of the Chi-Square test and neural networks for 100 breast cancer and 125 oral cancer patients. The Chi-Square classification has similar results to that of clinical examination in correlation of TNM classification. The artificial neural networks (MLP and RBF) provide more accuracy than the TNM staging system for using the TNM prognostic factors alone. Razi and Athappilly [32] study the comparative performance of the nonlinear regression, neural networks, and CART models to evaluate prediction accuracy for a continuous dependent variable and a set of binomial categorical predictor variables of a large dataset on smokers. They have used different prediction accuracy measuring procedures like Mean Absolute Percentage Error (MAPE), Mean Squared Error (MSE), and Large Prediction Error (LPE) to compare their performance and reported that the neural network and CART models predict better than the nonlinear regression model. Lin et al. [33] apply the neural network (multilayer perceptron (MLP)) on genetic data for the oral cancer detection.

Thus, we see that the data mining has not been optimally applied on oral cancer data to support the decision making process of practitioner towards the early detection and prevention of oral cancer. The exiting studies have the dataset too small or the numbers of attributes considered are limited. The linear regression and logistic regression have been used in literature, but with mainly two or three inputs [34–39]. The classification tree has also not explored much. The advanced data mining techniques, that is, artificial neural networks like multilayer perceptron (MLP) and radial basis function (RBF), have been applied by some researchers for prediction of oral cancer; however, other popular and more effective neural networks like cascade correlation neural network (CCNN), group method of data handling neural network (GMDH), and probabilistic neural network and general regression neural network (PNN/GRNN) have hardly been applied. Therefore, in this research work we have attempted to create PNN/GRNN model and compare it with other classification models build previously [40–44].

The various classification models developed are logistic regression analysis model [43], classification tree models like decision tree model, decision tree forest model, and TreeBoost model [40, 44], and artificial neural networks like multilayer perceptron (MLP) model [42], cascade correlation neural network (CCNN) model [41], probabilistic neural network and general regression neural network (PNN/GRNN) model [43]. The performance comparison of all the classification models developed using various data mining techniques is presented in Tables 6 and 7 for training and validation data, respectively.

Having presented the comprehensive comparison of all models, the best model for each estimation parameter is presented.  Table 8 presents the best model for each performance parameter for the training and validation data.

## 6. Conclusion

In this paper, we have discussed the probabilistic neural network and general regression neural network (PNN/GRNN) model for early diagnosis of disease, predicting the stage of the cancer, and chances of survivability of the oral cancer patients. This model can be of good help to the practitioners for improving the accuracy of the diagnosis and effectiveness of the treatment. Also, we have critically analyzed all data mining models and it has been observed that the probabilistic neural network and general regression neural network model displays competitive results for the training as well as validation data. The experimental results show that the probabilistic neural network and general regression neural network model displays the best classification accuracy, highest specificity and sensitivity, and better results in terms geometric mean of sensitivity and specificity, positive predictive value, negative predictive value, geometric mean of the PPV and NPV, average gain, precision, recall, *f*-measure, and area under ROC curve, among all the models, which makes it a robust model. Thus, the PNN/GRNN model is more suitable for predicting the survival rate of oral cancer patients.

## Figures and Tables

**Table 1:** Performance of PNN/GRNN model for classification of malignant and benign case for oral cancer.

Performance estimation parameter	Performance
Positive/negative ratio	3.0837
Accuracy	99.02%
True positive (TP)	75.02%
True negative (TN)	24.00%
False positive (FP)	0.49%
False negative (FN)	0.49%
Sensitivity	99.35%
Specificity	98.01%
Geometric mean of sensitivity-specificity	98.68%
Positive predictive value (PPV)	99.35%
Negative predictive value (NPV)	98.01%
Geometric mean of PPV and NPV	98.68%
Precision	99.35%
Recall	99.35%
*F*-measure	0.9935
Area under ROC curve	0.9974

**Table 2:** Performance of PNN/GRNN model for classification of various types of oral cancer.

Estimation parameter	Performance (in %)								
	Acantholytic	Adenocarcinoma	Basaloid	Lymphoepithelioma-like	Plaque-like	Sarcomatoid	SCC	Verrucous	Benign
Accuracy	99.61	99.41	99.71	99.90	99.90	99.61	99.95	95.61	100
True positive (TP)	0.10	0.10	0.10	0.00	0.00	0.10	56.20	1.37	35.90
True negative (TN)	99.51	99.32	99.61	99.90	99.90	99.51	37.76	94.24	64.10
False positive (FP)	0.00	0.00	0.00	0.00	0.00	0.00	6.05	0.10	0.00
False negative (FN)	0.39	0.59	0.29	0.10	0.10	0.39	0.00	4.29	0.00
Sensitivity	20.00	14.29	25	0.00	0.00	20.00	100	24.14	100
Specificity	100	100	100	100	100	100	86.19	99.90	100
Geometric mean of sensitivity and specificity	44.72	37.80	50	0.0	0.00	44.72	92.84	49	100
Positive predictive value (PPV)	100	100	100	0.00	99.90	100	90.28	93.33	100
Negative predictive value (NPV)	99.61	99.41	99.71	99.90	0.00	99.61	100	95.64	100
Geometric mean of PPV and NPV	99.80	99.71	99.85	0.00	0.00	99.80	95.02	94.48	100
Precision	100	100	100	0.00	0.00	100	90.28	93.33	100
Recall	20.00	14.29	25	0.00	0.00	20.00	100	24.14	100
*F*-measure	0.33	0.25	0.40	0.00	0.00	0.33	0.94	0.38	1.00

**Table 3:** Probability of occurrence of type of oral cancer using PNN/GRNN model.

Diagnosis (type of tumour)	Probability
Squamous cell carcinoma (SCC)	56.19%
Verrucous	5.6%
Acantholytic	0.48%
Basaloid	0.39%
Adenocarcinoma	0.68%
Sarcomatoid	0.48%
Lymphoepithelioma-like	0.09%
Plaque-like	0.09%
Benign	35.9%

**Table 4:** Performance of PNN/GRNN model for prediction of oral cancer stage.

Estimation parameter	Performance (in %)			
	Stage I	Stage II	Stage IV	Stage N0
Accuracy	99.90	86.93	86.83	100
True positive (TP)	0.00	7.90	43.02	35.90
True negative (TN)	99.90	79.02	43.80	64.10
False positive (FP)	0.00	0.00	13.17	0.00
False negative (FN)	0.10	13.07	0.00	0.00
Sensitivity	0.00	37.67	100.00	100.00
Specificity	100.00	100.00	76.88	100.00
Geometric mean of sensitivity-specificity	0.00	61.38	87.68	100.00
Positive predictive value	0.00	100.00	76.56	100.00
Negative predictive value	98.01	85.81	100.00	100.00
Geometric mean of PPV and NPV	0.00	92.62	87.50	100.00
Precision	0.00	100.00	76.56	100.00
Recall	0.00	37.67	100.00	100.00
*F*-measure	0.00	0.547	0.867	1.00

**Table 5:** Performance of PNN/GRNN model for predicting survivability of oral cancer patients.

Performance estimation parameter	Performance
Positive/negative ratio	0.674
Accuracy	69.95%
True positive (TP)	36.68%
True negative (TN)	33.27%
False positive (FP)	26.44%
False negative (FN)	3.61%
Sensitivity	91.04%
Specificity	55.72%
Geometric mean of sensitivity-specificity	71.22%
Positive predictive value (PPV)	58.11%
Negative predictive value (NPV)	90.21%
Geometric mean of PPV and NPV	72.41%
Precision	58.11%
Recall	91.04%
*F*-measure	0.709
Area under ROC curve	0.7491

**Table 6:** Comparison of performance of classification models for training data.

Estimation parameters	Linear regression	Decision tree	TreeBoost	MLP	CCNN	PNN/GRNN
Accuracy	60.10%	76.68%	74.76%	70.05%	72.10%	80.00%
True positive (TP)	1.37%	30.44%	32.80%	31.02%	33.46%	39.76%
True negative (TN)	68.73%	46.24%	41.95%	39.02%	38.63%	46.34%
False positive (FP)	0.98%	13.46%	17.80%	20.68%	21.07%	6.46%
False negative (FN)	38.93%	9.85%	7.44%	9.27%	6.83%	3.58%
Sensitivity	3.39%	75.54%	81.52%	77.00%	83.05%	92.78%
Specificity	98.37%	77.45%	70.20%	65.36%	64.71%	79.85%
Geometric mean of sensitivity and specificity	18.26%	76.49%	75.65%	70.94%	73.31%	80.55%
Positive predictive value (PPV)	58.33%	69.33%	64.82%	60.00%	61.36%	71.49%
Negative predictive value (NPV)	60.14%	82.43%	84.94%	80.81%	84.98%	90.79%
Geometric mean of PPV and NPV	59.23%	75.60%	74.20%	69.63%	72.21%	79.14%
Average gain for survival = *A*	1.25%	1.26%	1.369%	1.28%	1.31%	1.40%
Average gain for survival = *D*	1.34%	1.35%	1.57%	1.36%	1.43%	1.65%
Precision	58.33%	69.33%	64.82%	60.00%	61.36%	71.49%
Recall	3.39%	75.54%	81.52%	77.00%	83.05%	91.8%
*F*-measure	0.0641	0.7231	0.7221	0.6744	0.7058	0.7715
Area under ROC curve	0.722	0.835	0.8476	0.769	0.779	0.892

**Table 7:** Comparison of performance of classification models for validation data.

Estimation parameters	Linear regression	Decision tree	Decision tree forest	TreeBoost	MLP	CCNN	PNN/GRNN
Accuracy	61.27%	68.88%	67.41%	72.68%	69.76%	68.29%	73.76%
True positive (TP)	20.68%	25.85%	30.93%	32.30%	33.07%	30.34%	35.31%
True negative (TN)	40.59%	43.02%	36.49%	40.49%	36.68%	37.95%	41.88%
False positive (FP)	19.12%	16.68%	23.22%	19.02%	23.02%	21.79%	12.83%
False negative (FN)	19.61%	14.44%	9.37%	8.29%	7.22%	9.95%	4.41%
Sensitivity	51.33%	64.16%	76.76%	79.52%	82.08%	75.30%	87.67%
Specificity	67.97%	72.06%	61.11%	68.03%	61.44%	63.56%	69.46%
Geometric mean of sensitivity and specificity	59.07%	68.00%	68.49%	73.55%	71.01%	69.18%	74.05%
Positive predictive value (PPV)	51.96%	60.78%	57.12%	62.86%	58.96%	58.24%	62.86%
Negative predictive value (NPV)	67.42%	74.87%	79.57%	83.00%	83.56%	79.23%	88.17%
Geometric mean of PPV and NPV	59.19%	67.46%	67.42%	72.23%	70.19%	67.93%	72.23%
Average gain for survival = *A*	1.149	1.15	1.273	1.274	1.28	1.26%	1.32%
Average gain for survival = *D*	1.17	1.17	1.324	1.413	1.31	1.32%	1.48%
Precision	51.96%	60.78%	57.12%	62.86%	58.96%	58.24%	63.53%
Recall	51.33%	64.16%	76.76%	79.52%	82.08%	75.30%	86.67%
*F*-measure	0.5164	0.6243	0.655	0.7021	0.6862	0.6568	0.6593
Area under ROC curve	0.631	0.835	0.765	0.7705	0.739	0.731	0.821

**Table 8:** Performance parameter-wise best model for training and validation data.

Estimation parameters	Description	Training data		Validation data	
		Model	%	Model	%
Accuracy	Accuracy of classification	PNN/GRNN	80.00%	PNN/GRNN TreeBoost	73.76% 72.68%

True positive (TP)	Patients who are predicted as malignant among the malignant patients	PNN/GRNN	39.76%	PNN/GRNN MLP	35.51% 33.07%

True negative (TN)	Patients who are predicted as nonmalignant among nonmalignant patients	PNN/GRNN	46.34%	Decision tree PNN/GRNN	43.02% 41.88%

False positive (FP)	Patients who are predicted as malignant among nonmalignant patients	PNN/GRNN	3.58%	PNN/GRNN	12.83%

False negative (FN)	Patients who are predicted as nonmalignant among malignant patients	PNN/GRNN	3.58%	PNN/GRNN MLP	4.41% 7.22%

Sensitivity	Probability to correctly predict malignancy	PNN/GRNN	92.78%	PNN/GRNN MLP	87.67% 82.08%

Specificity	Probability to correctly predict nonmalignant cases	PNN/GRNN	79.85%	Decision tree PNN/GRNN	72.06% 69.46%

Geometric mean of sensitivity and specificity	Geometric mean of sensitivity and specificity	PNN/GRNN	80.55%	PNN/GRNN TreeBoost	74.05% 73.55%

Positive predictive value (PPV)	Proportion of patients with the disease who are correctly predicted to have the disease	PNN/GRNN	71.49%	PNN/GRNN TreeBoost	62.86% 62.86%

Negative predictive value (NPV)	Proportion of patients who do not have the disease and who are correctly predicted as not having the disease	PNN/GRNN	90.79%	PNN/GRNN MLP	88.17% 83.56%

Geometric mean of PPV and NPV	Geometric mean of PPV and NPV	PNN/GRNN	79.14%	PNN/GRNN TreeBoost	72.23% 72.23%

Average gain for survival = *A*	The gain shows how much of an improvement is provided by the model	PNN/GRNN	1.40%	PNN/GRNN	1.32%

Average gain for survival = *D*	The gain shows how much of an improvement is provided by the model	PNN/GRNN	1.65%	PNN/GRNN	1.48%

Precision	Proportion of cases selected by the model that have the true value; precision is equal to PPV	PNN/GRNN	71.49%	TreeBoost PNN/GRNN	62.86% 63.53%

Recall	Proportion of the true cases that are identified by the model; recall is equal to sensitivity	PNN/GRNN GMDH	91.8% 91.04%	PNN/GRNN MLP	86.67% 82.08%

*F*-measure	It combines precision and recall to give an overall measure of the quality of the prediction	PNN/GRNN	0.7715	TreeBoost PNN/GRNN	0.7021 0.6593

Area under ROC curve	Area under the Receive Operating Characteristic (ROC) curve for the model	PNN/GRNN	0.892	Decision Tree PNN/GRNN	0.835 0.821

## References

[1] Parkin D. M., Laara E., Muir C. S. (1988). Estimates of the worldwide frequency of sixteen major cancers in 1980. *International Journal of Cancer*.

[2] Langdon J. D., Russel R. C., Williams N. S., Bulstrode Arnold C. J. K. (2000). *Oral and Oropharyngeal Cancer Practice of Surgery*.

[3] Jemal A., Siegel R., Xu J., Ward E. (2010). Cancer statistics, 2010. *CA: A Cancer Journal for Clinicians*.

[4] Sankaranarayanan R., Masuyer E., Swaminathan R., Ferlay J., Whelan S. (1998). Head and neck cancer: a global perspective on epidemiology and prognosis. *Anticancer Research*.

[5] Ferlay J., Shin H.-R., Bray F., Forman D., Mathers C., Parkin D. M. (2010). Estimates of worldwide burden of cancer in 2008: GLOBOCAN 2008. *International Journal of Cancer*.

[6] Sankaranarayanan R., Ramadas K., Thomas G. (2005). Effect of screening on oral cancer mortality in Kerala, India: a cluster-randomised controlled trial. *The Lancet*.

[7] Dinshaw K. A., Ganesh B. (2008). *Annual Report 2002–2005, Hospital Based Cancer Registry*.

[8] Elango J. K., Gangadharan P., Sumithra S., Kuriakose M. A. (2006). Trends of head and neck cancers in Urban and rural India. *Asian Pacific Journal of Cancer Prevention*.

[9] Manoharan N., Tyagi B. B., Raina V. (2010). Cancer incidences in rural Delhi—2004-05. *Asian Pacific Journal of Cancer Prevention*.

[10] Agrawal M., Pandey S., Jain S., Maitin S. (2012). Oral cancer awareness of the general public in Gorakhpur city, India. *Asian Pacific Journal of Cancer Prevention*.

[11] Khandekar P. S., Bagdey P. S., Tiwari R. R. (2006). Oral cancer and Some epidemiological factors: a hospital based. *Indian Journal of Community Medicine*.

[12] Chakrabarti S., Ester M., Fayyad U. Data mining curriculum.

[13] Fayyad U., Piatetsky-Shapiro G., Smyth P. (1996). From data mining to knowledge discovery in databases. *AI Magazine*.

[14] Fayyad U. M., Piatetsky-Shapiro G., Smyth P. (1996). From data mining to knowledge discovery: an overview. *Advances in Knowledge Discovery and Data Mining*.

[15] Christopher C. (2010). *Encyclopædia Britannica: Definition of Data Mining*.

[16] Hastie T., Tibshirani R., Friedman J. (2009). *The Elements of Statistical Learning: Data Mining, Inference, and Prediction*.

[17] Han J., Kamber M., Pei J. (2011). *Data Mining: Concepts and Techniques*.

[18] Sharma N., Om H. (2012). Framework for early detection and prevention of oral cancer using data mining. *International Journal of Advances in Engineering and Technology*.

[19] Forman G. (2003). An extensive empirical study of feature selection metrics for text classification. *Journal of Machine Learning Research*.

[20] Peng H. C., Long F., Ding C. (2005). Feature selection based on mutual information: criteria of max-dependency, max-relevance, and min-redundancy. *IEEE Transactions on Pattern Analysis and Machine Intelligence*.

[21] Brown G., Pocock A., Zhao M.-J., Lujan M. (2012). Conditional likelihood maximisation: a unifying framework for information theoretic feature selection. *Journal of Machine Learning Research*.

[22] Senliol B., Gulgezen G., Yu L., Cataltepe Z. Fast Correlation Based Filter (FCBF) with a different search strategy.

[23] http://www.dtreg.com/.

[24] Specht D. F. (1990). Probabilistic neural networks. *Neural Networks*.

[25] Specht D. F. Enhancements to probabilistic neural networks.

[26] Wilson D. R., Martinez T. R. (1998). Improved center point selection for probabilistic neural networks. *Artificial Neural Nets and Genetic Algorithms: Proceedings of the International Conference in Norwich, U.K., 1997*.

[27] Smith A. E., Mason A. K. (1997). Cost estimation predictive modeling: regression versus neural network. *The Engineering Economist*.

[28] Bruins H. H., Koole R., Jolly D. E. (1998). Pretherapy dental decisions in patients with head and neck cancer: a proposed model for dental decision support. *Oral Surgery, Oral Medicine, Oral Pathology, Oral Radiology, and Endodontics*.

[29] Rosmai M. D., Sameemii A. K., Basir A., Mazlipahiv I. S., Norzaidi M. D. (2010). The use of artificial intelligence to identify people at risk of oral cancer: empirical evidence in Malaysian University. *International Journal of Scientific Research in Education*.

[30] Rosmai M. D., Basir A., Kareem S. A., Ismail S. M., Norzaidi M. D. (2012). Determining the critical success factors of oral cancer susceptibility prediction in Malaysia using fuzzy models. *Sains Malaysiana*.

[31] HariKumar R., Vasanthi N. S., Balasubramani M. (2012). Performance analysis of artificial neural networks and statistical methods in classification of oral and breast cancer stages. *International Journal of Soft Computing and Engineering*.

[32] Razi M. A., Athappilly K. (2005). A comparative predictive analysis of neural networks (NNs), nonlinear regression and classification and regression tree (CART) models. *Expert Systems with Applications*.

[33] Lin H.-S., Talwar H. S., Tarca A. L. (2007). Autoantibody approach for serum-based detection of head and neck cancer. *Cancer Epidemiology Biomarkers and Prevention*.

[34] Sunny L., Yeole B. B., Hakama M. (2004). Oral cancers in mumbai, india: a fifteen years perspective with respect to incidence trend and cumulative risk. *Asian Pacific Journal of Cancer Prevention*.

[35] Arulchinnappan S., Karunakaran K., Rajendran G. (2010). A survey of oral cancer using fuzzy linear regression algorithm. *Austral-Asian Journal of Cancer*.

[36] Arulchinnappan S., Karunakaran K., Rajendran G. (2011). Deduction of oral cancer using fuzzy linear regression. *Journal of Computer Science*.

[37] Harty L. C., Caporaso N. E., Hayes R. B. (1997). Alcohol dehydrogenase 3 genotype and risk of oral cavity and pharyngeal cancers. *Journal of the National Cancer Institute*.

[38] Hung H.-C., Chuang J., Chien Y.-C. (1997). Genetic polymorphisms of CYP2E1, GSTM1, and GSTT1; environmental factors and risk of oral cancer. *Cancer Epidemiology Biomarkers and Prevention*.

[39] Balaram P., Sridhar H., Rajkumar T. (2002). Oral cancer in Southern India: the influence of smoking, drinking, paan-chewing and oral hygiene. *International Journal of Cancer*.

[40] Sharma N., Om H. (2013). Data mining models for predicting oral cancer survivability. *Network Modeling Analysis in Health Informatics and Bioinformatics*.

[41] Sharma N., Om H. (2014). Cascade correlation neural network model for classification of oral cancer. *WSEAS Transactions on Biology and Biomedicine*.

[42] Sharma N., Om H. (2014). Using MLP and SVM for predicting survival rate of oral cancer patients. *Network Modeling Analysis in Health Informatics and Bioinformatics*.

[43] Sharma N., Om H. Predicting oral cancer survivability using LR and PNN/GRNN models.

[44] Sharma N., Om H. (2014). Predictive data mining for oral cancer. *Case Studies in Intelligent Computing—Achievements and Trends*.

